# Acute Motor Axonal Neuropathy in Association with Hepatitis E

**DOI:** 10.3389/fneur.2018.00062

**Published:** 2018-02-09

**Authors:** Araz Al-Saffar, Bassam Al-Fatly

**Affiliations:** ^1^Department of Internal Medicine, College of Medicine, Al-Nahrain University, Baghdad, Iraq; ^2^Department of Neurology, Movement Disorders and Neuromodulation Unit, Charité – Universitätsmedizin Berlin, corporate member of Freie Universität Berlin, Humboldt-Universität zu Berlin, and Berlin Institute of Health, Berlin, Germany

**Keywords:** Guillain–Barré syndrome, acute motor axonal neuropathy, hepatitis E, muscle weakness, nerve conduction study

## Abstract

Guillain–Barré syndrome (GBS) is an acute peripheral neuropathy that develops as a result of post-infectious immune-mediated nerve injury. It can be classified into classic and variant GBS. Acute motor axonal neuropathy (AMAN) is a subtype of GBS with the key clinical features of pure motor weakness, areflexia, absence of sensory symptoms, and lack of neurophysiologic evidence of demyelination. We reported a case of acute motor axonal neuropathy in association with hepatitis E infection. A young woman was referred to us after a period of nausea, fever, and diarrhea. She had unexplained muscle weakness at admission and has been diagnosed with acute hepatitis E infection. A rigorous clinical neurological assessment revealed bilateral symmetrical weakness, which affects the lower limbs more than the upper limbs, with no evidence of sensory involvement. Neurophysiological measurements indicated acute axonal injury without clues to demyelination. A diagnosis of acute motor axonal neuropathy subtype has been made, to which she only received supportive therapy. The symptoms resolved spontaneously and full recovery of motor function was attained after 35 days of weakness onset with complete normalization of neurophysiologic parameters.

## Introduction

Guillain–Barré syndrome (GBS) is an acute peripheral neuropathic disease characterized by ascending flaccid paralysis of symmetrical distribution that can present with motor and/or sensory symptoms (1). GBS comprises the most common form of acute inflammatory demyelinating neuropathy (AIDP) as well as other variants like Miller Fisher syndrome, acute motor and sensory axonal neuropathy, and acute motor axonal neuropathy (2).

Guillain–Barré syndrome is caused by post-infectious immunological modulation that leads to immunologically mediated damage of peripheral nerve parts (myelin, axon, or both) which may present with mixed sensorimotor or pure motor symptoms. Hepatitis viruses are among the emerging pathogens that could be encountered in the context of GBS (1). Specifically, hepatitis E infection was described in association with classical GBS or its variants, although none of the latter has been confirmed to lie in the pure motor category of GBS (3–7).

In our case report, we describe a woman with acute pure motor subtype of GBS that has developed in association with hepatitis E infection and recovered spontaneously in parallel with liver functions normalization.

## Case Report

A 19-year-old Iraqi woman sought care for bilateral lower limb weakness without sensory symptoms during her 28th week of pregnancy. The patient suffered 3 days of fever associated with nausea, decreased appetite, and diarrhea. One day after fever cessation, she developed bilateral lower limb weakness. She has been admitted to our hospital 24 h after the onset of lower limb weakness for further evaluation. Her initial blood work-up revealed hypokalemia for which she was thought to have developed weakness and received intravenous potassium-containing fluids. Weakness failed to improve despite normalization of serum potassium level. 3 days after the onset of weakness, she has been referred to us for further neurological assessment. At this point, she noticed that her lower limbs weakness has increased. Additionally, her upper limbs started to develop mild weakness. The patient reported uneventful pregnancy and no history of drugs intake. She had no travel history or contact with people suffering from similar symptoms. She reported eating from street food vendors.

Neurological examination revealed a modified MRC muscle power grade of 2/5 for dorsiflexors, plantar flexors, and knee flexors and extensors, 3/5 for hip flexors and abductors, and −4/5 for finger intrinsic muscles, wrist flexors and extensors, elbow flexors and extensors, and shoulder abductors. Her lower limb deep tendon reflexes were bilaterally absent. No evidence of cranial nerves or cerebellar involvement has been noted. In addition, her sensory examination was completely normal. Her vital signs were normal. General physical examination was evident for conjunctival icterus. Tender hepatomegaly was noted during abdominal examination. Her cardiac and respiratory functions were normal.

Laboratory investigation showed normal complete blood count and ESR. Her serum electrolytes, blood urea, and serum creatinine were normal. Liver function tests showed elevated total serum bilirubin level (2.8 mg/dL, reference 0.2−1.2 mg/dL), serum alanine transaminase (ALT = 829 U/L, reference 5−34 U/L), and aspartate transaminase (AST = 471 U/L, reference 0−55 U/L). Serum ceruloplasmin level was normal. Deranged coagulation profile is also evident [Prothrombin time (PT) = 16 s, normal range: up to 13 s; activated Partial Thromboplastin time (aPTT) = 41, normal range: up to 36 s] along with reduced serum albumin level (2.2 g/dL, normal range: 3.5−5.2 g/dL). Hepatitis E antibodies assessment using enzyme-linked immunosorbent assay method revealed elevated level of IgM (25.7 IU/ml, normal <2 IU/ml) as well as IgG level (15.9 IU/ml, normal <2 IU/ml). General stool examination revealed liquid feces with no evidence of blood or pus. Serological testing was negative for hepatitis A, B, and C, cytomegalovirus, Epstein–Barr virus, and human immunodeficiency virus. Serum antinuclear antibody (ANA) and thyroid function test were normal. We have been hindered from doing a lumbar puncture and cerebrospinal fluid analysis because of the bleeding tendency revealed by prolonged PT and PTT. Additionally, the patient refused to undergo a lumbar puncture procedure. Patient’s abdominal and pelvic ultrasonographic examination revealed hepatomegaly.

Initial nerve conduction study (NCS) (7 days after the onset of weakness), showed evidence of axonal motor neuropathy of symmetrical distribution affecting the lower limbs more than the upper limbs with normal sensory NCS parameters. Needle Electromyography (EMG) study decreased recruitment pattern, consistent with acute onset neurogenic process. Repetitive nerve stimulation targeted to detect deficit associated with periodic paralysis was normal.

These findings were consistent with a diagnosis of acute motor axonal neuropathy variant of GBS in association with hepatitis E infection (as indicated by the clinical scenario, deranged liver function test, and serological evidence of hepatitis E antibody). The patient has been given no specific treatment except for close observation and supportive measures. Repeated NCS/EMG after 35 days of weakness onset (see Table 1) showed full recovery of motor neurophysiological parameters. These changes have been paralleled by clinical improvement in weakness and normalization of liver function test values.

**Table 1:** Comparison of motor nerve conduction study (NCS) results at onset and resolution of motor weakness.

Parameters	R. sural	L. sural	R. peroneal	L. peroneal	R. tibial	L. tibial	R. median	L. median	R. ulnar	L. ulnar
**Initial NCS (7 days after onset of weakness)**										

dCMAP amp. (mV)			0.5	0.7	2.1	1.9	3.8	4.0	3.2	3.4
Normal values (mV)			>2.0		>4.0		>4.4		>6.0	
DML (ms)			4.2	4.1	3.0	3.1	2.4	2.5	2.46	2.75
Normal values (ms)			<6.5		<5.8		<4.2		<3.1	
MCV (m/s)			51.7	50.7	49.1	50.1	58.8	57.0	54.7	54.0
Normal values (m/s)			>40		>40		>50		>50	
SNAP amp. (μV)	14	15					23	22	21	24
Normal values (μV)	>10						>20		>18	
SCV (m/s)	55.2	54.3					63.3	61.2	60.3	60.9
Normal values (m/s)	>36						>45		>44	

**Resolution of motor weakness (35 days after weakness onset)**										

dCMAP amp. (mV)			4.3	4.1	4.3	4.7	5.0	5.3	4.9	5.1
DML (ms)			3.7	3.9	2.6	3.0	2.3	2.4	2.6	2.9
CV (m/s)			57.7	58.8	50.2	51.0	58.9	56.9	52.2	52.1
SNAP amp. (μV)	16	13					24	21	23	-
SCV (m/s)	53.5	52.1					62.5	60.8	59.4	-

*R, right; L, left; dCMAP, distal compound muscle action potential amplitude; MCV, motor conduction velocity; SCV, sensory conduction velocity; SNAP, sensory nerve action potential*.

## Discussion

Hepatitis E infection is considered among the most common feco-orally transmitted hepatitis in developing countries (8). Many case reports have documented the association of hepatitis E infection with a variety of neurological manifestations including encephalitis and different types of neuropathies (9, 10). Of importance are those with reported peripheral neuropathic diseases like neuralgic amyotrophy, ataxic neuropathy, and AIDP or GBS (11, 12). Furthermore, some of GBS variants, like Miller Fisher syndrome and acute motor and sensory Axonal variant, have been reported by others (5, 12).

Pure motor or mixed sensorimotor fibers involvement categorizes GBS into different subtypes, with the underlying pathologic mechanisms being either demyelination or axonopathy (1). AMAN is a subtype of GBS characterized by pure motor involvement and absence of evidence of demyelination in electrophysiological tests (2).

Acute motor axonal neuropathy was linked to *Campylobacter jejuni* and Zika virus infection in the literatures (13, 14). There is uncertainty about the frequency with which AMAN was reported and this was claimed to be as a consequence of variability in infectious agent exposure according to the geographical areas. Nevertheless, AMAN has an estimated incidence of 30–65% in Asia and Central and South America (1).

To our best knowledge, this is the first case report of AMAN that has developed in association with acute hepatitis E infection in an immunocompetent pregnant woman and has recovered rapidly and spontaneously. Van den Berg et al. have mentioned a single case out of 201 GBS cases in the Netherlands, in which neurophysiologic evidence of AMAN has been documented in the presence of IgM anti-HEV antibody (15). However, the recovery pattern was prolonged and the treatment status is unknown. In our case, the diagnosis of hepatitis E infection was based on finding of high hepatitis E IgM in addition to rising level of IgG to greater than fivefolds. We did not determine viral RNA load using polymerase chain reaction-reverse transcriptase technique. Nevertheless, hepatitis E antibodies profile sufficed for the diagnosis of acute hepatitis E infection (16). Needless to say, serum transaminases profile (ALT is higher elevated than AST) does agree with a predominant hepatocellular injury, among which, hepatotropic infection is a common type (17, 18). Arguing against the diagnosis of viral hepatitis is also a possibility; however, the lack of evidence of alternative diagnosis of hepatitis like drug induced, alcoholic, or immune hepatitis could be excluded by lack of history of drug or alcohol intake and laboratory clues of immune hepatitis. Based on the clinical and neurophysiological features, the patient developed an acute motor peripheral neuropathy of ascending pattern with no clues to sensory involvement. Absence of alternative diagnosis was confirmed by absence of indicators of vasculitic neuropathy (normal ESR and ANA) or possible myasthenia gravis or periodic paralysis (normal repetitive nerve stimulation study). We collected enough evidence to reach level 2 diagnostic certainties according to Brighton’s criteria for diagnosing GBS (19).

Although previous case reports showed presence of anti-GM1 and anti-GM2 anti-ganglioside antibodies in cases of GBS preceded by hepatitis E infection (20–22), we could not catch the opportunity to report anti-ganglioside antibodies in our case due to their unavailability as routine tests. Furthermore, anti-ganglioside antibodies are not required for the diagnosis of AMAN according to Brighton’s criteria (19).

Hiraga et al. described a rapid recovery pattern in 27% of 44 cases of AMAN (23). All these cases required treatment with either immunoglobulin or plasmapheresis and none showed a spontaneous recovery pattern. Our case scenario fits into the rapid pattern of recovery. Additionally, the recovery was complete and spontaneous with no need for any specific therapeutic intervention. Rapid and spontaneous recovery has been formerly reported in a case of AIDP associated with hepatitis E infection (6). This would suggest that the presence of hepatitis E is an indicator of favorable recovery pattern in the context of AMAN.

The mechanism by which hepatitis E virus can cause neurological injury is not completely understood. It has been suggested that viral molecular mimicry to a neural antigen could serve to enhance immunological reaction (1, 2). In our case, it seems that rapid recovery could be the result of clearance of the viral molecular particle, which presumably mimics axonal protein, by the immune system.

## Concluding Remarks

The number of causative pathogens associated with GBS and its variants are expanding. We reported a case of AMAN variant of GBS in association with acute hepatitis E virus infection. This case highlights the importance of diagnosing AMAN in the context of hepatitis E and encourages further research to underpin the mechanism of axonal injury in AMAN variant. It also underscores the favorable, spontaneous recovery of AMAN in association with acute hepatitis E infection.

## Ethics Statement

Written informed consent to publish the report was obtained from the patient.

## Author Contributions

AA-S was involved with the care of the patient, clinical diagnosis, treatment, and revision of the manuscript. BA-F was also involved in the diagnosis of the condition (conducted and interpreted neurophysiological tests), initial write up, editing, and revision the manuscript.

## Conflict of Interest Statement

The authors declare that the research was conducted in the absence of any commercial or financial relationships that could be construed as a potential conflict of interest. The reviewer CT and handling editor declared their shared affiliation.
